# Radiation-Induced Oropharyngeal Squamous Cell Carcinoma: Case Report and Review of the Literature

**DOI:** 10.3390/curroncol30070492

**Published:** 2023-07-14

**Authors:** Lorenzo Giannini, Andrea Alliata, Valentina Cristofaro, Fabiola Incandela, Madia Pompilio, Arianna Ottini, Stefano Cavalieri, Imperia Nuzzolese, Nicola Alessandro Iacovelli, Marzia Franceschini, Alberto Deganello

**Affiliations:** 1 Department of Otorhinolaryngology, Maxillofacial, and Thyroid Surgery, Fondazione IRCCS Istituto Nazionale dei Tumori di Milano, 20133 Milan, Italy; 2 Department of Clinical Sciences and Community Health, University of Milan, 20122 Milan, Italy; 3 Head and Neck Medical Oncology Department, Fondazione IRCCS Istituto Nazionale dei Tumori di Milano, 20133 Milan, Italy; 4 Department of Oncology and Hemato-Oncology, University of Milan, 20122 Milan, Italy; 5 Radiotherapy Department, Fondazione IRCCS Istituto Nazionale dei Tumori di Milano, 20133 Milan, Italy

**Keywords:** radiotherapy, radiation-induced cancer, second primary tumour, head and neck, multidisciplinary team

## Abstract

Background: Radiation therapy (RT) is a mainstay for the treatment of head and neck (HN) cancers, with 80% of patients receiving such treatment. Radiation-induced malignancies represent a life-threatening long-term effect of RT, with an incidence of 0.5% to 15%. Case Description: After 13 years, a 33-year-old woman treated with chemo-radiotherapy for nasopharyngeal carcinoma developed a locally advanced, radiation-induced, p16-negative oropharyngeal squamous cell carcinoma (SCC) at the base of the tongue. Chemo/immunotherapy was administered as a first-line treatment. Given the optimal response and the feasibility of surgery, after three cycles, the patient underwent a total glossectomy, bilateral neck dissection, and reconstruction with a thoraco-dorsal free flap. A histological examination found SCC with a residual cancer burden of 70% and free margins. Discussion: The mechanisms responsible for carcinogenesis after RT are still not completely clear. Diagnosis may be challenging due to the previous treatment; growth patterns are unusual, and lymphotropism is lower. Prognosis is usually poor since surgical resectability is often not achievable. Conclusions: Radiation-induced malignancies are difficult to treat. Patient management should always be discussed at a multidisciplinary level. Future research is needed to assess whether the promising results of clinical studies with pre-operative immunotherapy in locally advanced HN SCC patients may be translated into radiation-induced cancers.

## 1. Introduction

Head and neck malignancies represent some of the most common cancers worldwide, with an annual incidence of more than 900,000 cases, approximately 400,000 deaths, and a global tendency to increase [1,2]. Among a wide variety of histological subtypes, head and neck squamous cell carcinoma (HNSCC) is the most common entity, accounting for 90% of all head and neck malignancies [3].

In recent decades, radiation therapy (RT), alone and as part of a multimodal approach, has confirmed its role as a cornerstone in the treatment of head and neck tumours in both adjuvant and in radical settings, improving the life expectancy of patients; it is estimated that 80% of patients affected by a head and neck malignancy will receive RT [4]. As survival improves, the late effects of RT can affect long-term health. The most common effects are represented by xerostomia, dysphagia, fatigue, and trismus, as well as neurologic complications such as hearing loss, convulsions, and cranial nerve palsy [5,6]. In addition, RT has been found to increase the risk of secondary malignancies. The carcinogenic role of irradiation has been described since the beginning of the 20th century, leading to the identification of a new pathological entity called radiation-induced malignancy (RIM). To be defined as such, the RIM should satisfy the following criteria: it must arise within an irradiated field, and its histology must be different from the treated tumour, with no presence of the new mass during irradiation and with an onset latency of at least 3 years from the index tumour treatment [7,8].

The present article will describe a case of oropharyngeal radiation-induced squamous cell carcinoma (RISCC) and will discuss it in view of the recent literature.

## 2. Detailed Case Description

A 33-year-old woman with a history of cT3 cN3 cM0 EBV-related, undifferentiated nasopharyngeal carcinoma (NPC), diagnosed in 2009, was treated with induction chemotherapy—three cycles of a 5-fluorouracil, cisplatin, and docetaxel (TPF) regimen—followed by concurrent chemo-radiotherapy (with a cumulated platinum dose of 300 mg/mq). Intensity-modulated radiation therapy (IMRT) step-and-shoot was employed to deliver a total dose of 70 Gy in 35 fractions to the nasopharynx and bilateral upper neck levels, while the bilateral lower neck levels received 54 Gy in 30 fractions. The patient achieved a complete response to treatment. There was no evidence of disease during follow-up.

After 13 years, the woman sought medical attention, complaining about the onset of a swelling in the left posterior part of the tongue. Her clinical history was unremarkable except for previous NPC; the patient never smoked and denied alcohol consumption.

**Figure 1:**
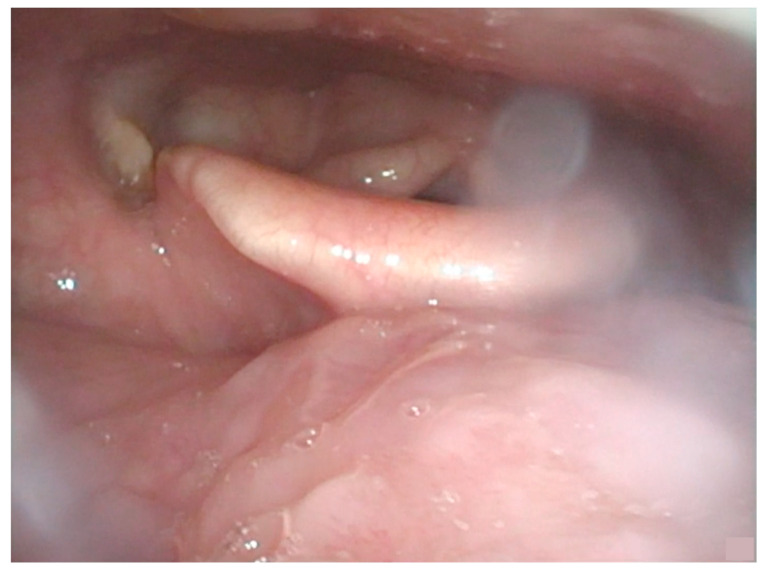
Endoscopic view showing submucosal bulging of the left base of the tongue, without ulceration. Tumour passes through the midline. There is no laryngeal or hypopharyngeal infiltration.

A physical examination with fibre-optic endoscopy showed a bulging of the left base of the tongue with no mucosal ulceration; upon palpation, the submucosal lesion extended superiorly until the amigdaloglossus sulcus, laterally to the caudal half of the left tonsil, and caudally towards the right vallecula without reaching its bottom, medially crossing approximately 1 cm over the midline (Figure 1).

**Figure 2:**
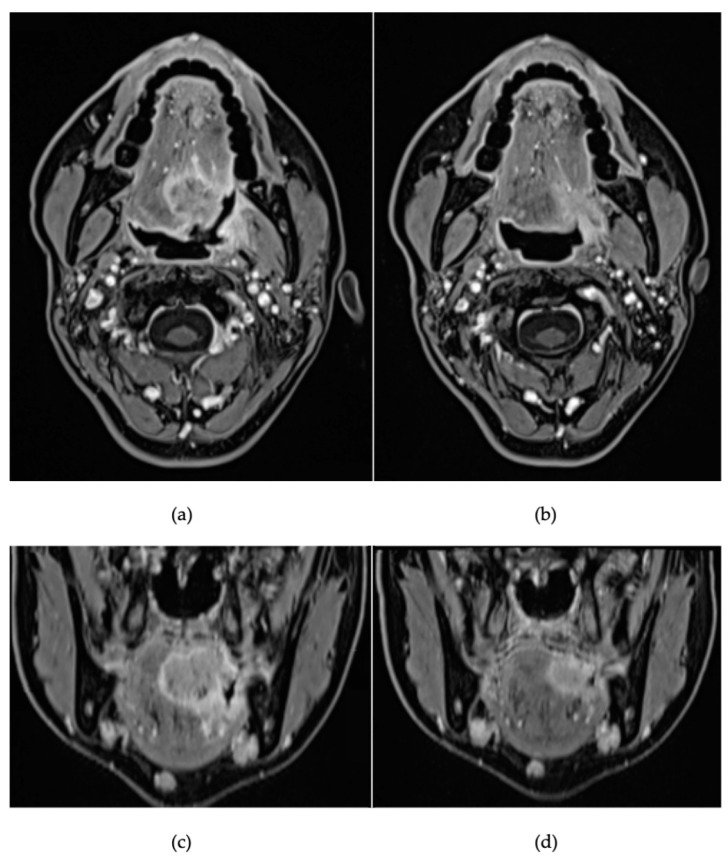
MRI axial and coronal sections before (respectively (**a**,**c**)) and after (**b**,**d**) chemo-immunotherapy. Images show a global reduction of the tumour (24 × 18 mm vs. 36 × 30 mm); the lesion still involves the tonsillar region and palatoglossal muscle, with milder involvement of the hyoglossus muscle. No variations in the submandibular and submental lymph nodes’ dimension and morphology are reported.

Magnetic resonance imaging (MRI) showed a pathologic hyperintense area of 30 × 36 mm (anteroposterior × craniocaudal), involving the left base of tongue, with the invasion of intrinsic and extrinsic tongue muscles, extension beyond the median septum and into the palatoglossus muscle (Figure 2), with suspect ipsilateral lymph nodes at level IA and IIA and bilateral IB levels, with no evidence of extracapsular extension.

Positron emission tomography/computed tomography (PET/CT) with ^18^F-fluorodeoxyglucose (FDG) confirmed pathological metabolic activity only in the primary lesion and in one ipsilateral node at level IIa.

Incisional biopsy was positive for moderately differentiated (G2) squamous cell carcinoma (SCC), p16-negative, with a programmed death-ligand 1 (PD-L1) combined positive score (CPS) > 20 (22). PD-L1 immunohistochemistry was performed on 3 μm thick formalin-fixed, paraffin-embedded (FFPE) sections via the 22C3 PharmDx assay (mouse monoclonal primary anti-PD-L1 antibody, Dako, Carpinteria, CA, USA), using an Autostainer Link 48 (Agilent Technologies, Santa Clara, CA, USA). The immunostains were evaluated by expert head and neck pathologists, and the combined positive sore (CPS), defined as the number of PD-L1-positive tumour cells, lymphocytes, and macrophages divided by the total number of viable tumour cells and multiplied by 100, was determined following their recommendations.

According to the AJCC TNM, 8th edition, the tumour was cT4a cN2c cM0—Stage IVA.

After a multidisciplinary discussion, the tumour was considered amenable to total glossectomy with laryngeal preservation, bilateral neck dissection, and reconstruction with a free flap. However, due to the advanced stage, the entity of the surgery, and the previous radiation therapy, the multidisciplinary team (MDT) decided to administer first-line systemic treatment chemo/immunotherapy. After discussion with the patient, the potentiality of salvage surgery was postponed after assessing the response to first-line therapy.

Given the CPS > 20, the patient received the following chemo-immunotherapy: pembrolizumab 200 mg q21 + cisplatin 100 mg/m^2^ q21 + 5-fluorouracil 1000 mg/m^2^/day × 4 days q21 (the baseline assessment of dihydropyrimidine dehydrogenase polymorphisms did not reveal any mutant variants associated with an increased risk of fluoropyrimidine-related toxicities). Due to cisplatin-related ototoxicity and the cumulative dose received, cisplatin was ceased from the second cycle in favour of carboplatin AUC4 q21. No further complications occurred during the treatment, and the patient received a total of three courses of chemo-immunotherapy.

Disease re-assessment was performed after the third cycle via MRI and PET-CT scans. These exams showed a partial radiological response (max diameter of 18 mm vs. 30 mm, Figure 2) and metabolic activity reduction (SUV max of 10.5 vs. 17.5). No significant nodal reduction was observed in MRI, but lymph node uptake was no longer detected on PET-CT.

An endoscopic examination under narcosis revealed a large ulceration replacing the original submucosal tongue base bulge that did not extend over the midline; new biopsies confirmed the presence of disease. Given the optimal response to first-line treatment and the feasibility of salvage surgery, after an MDT discussion, the patient was operated on.

**Figure 3:**
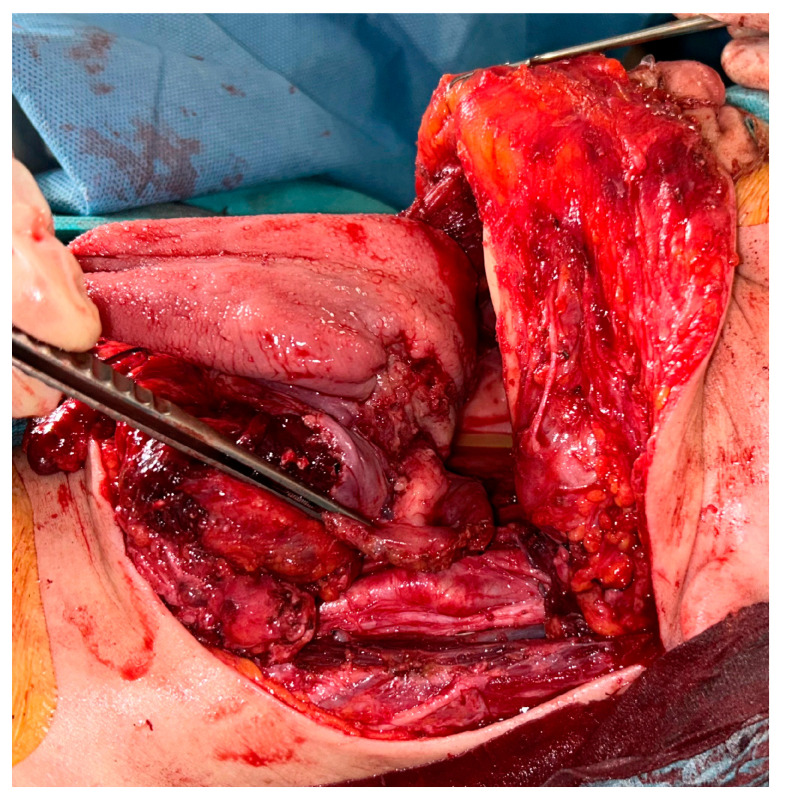
Intraoperative view of pull-through total glossectomy: an ulcerating lesion of the base of the tongue replacing the previous bulging can be seen; resection was extended to the left tonsil (between the forceps).

**Figure 4:**
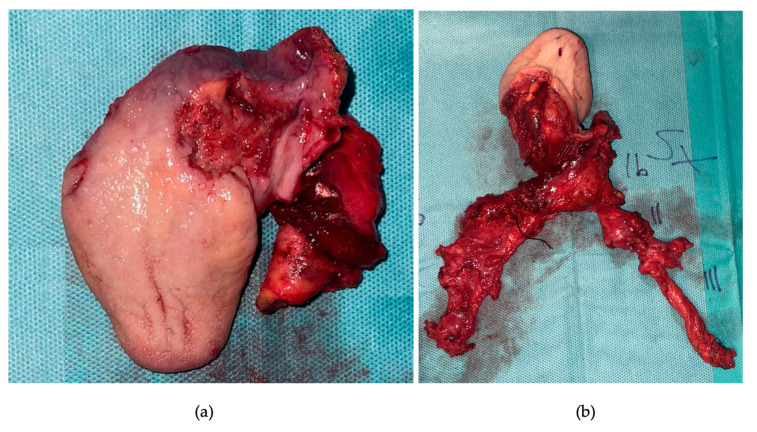
Surgical specimen: (**a**) close view of the tumour, with evidence of macroscopic invasion of left amigdaloglossus sulcus, vallecula, and tonsillar lodge; (**b**) view of the en bloc resection.

Following tracheotomy, the patient underwent a pull-through total glossectomy with extension to the whole tonsillar fossa and the left floor of mouth, en bloc with ipsilateral selective neck dissection of levels I-IV and contralateral selective neck dissection of levels I-III (Figure 3 and Figure 4).

**Figure 5:**
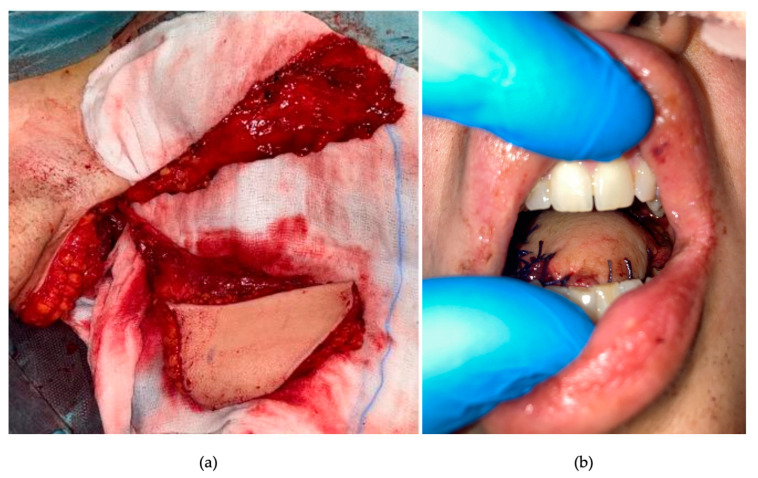
(**a**) Intraoperative view of the chimeric free-flap, harvested on the right thoraco-dorsal pedicle; (**b**) final intraoperative reconstructive result.

A latissimus dorsi myo-fascio-cutaneous free flap with a chimeric fascial component from the serratus anterior muscle [9] was simultaneously harvested in a supine position during tumour resection. The myocutaneous latissimus dorsi created the new tongue, while the chimeric fascial component was used to resurface the left tonsillar fossa (Figure 5).

The post-operative course was uneventful: the tracheotomy tube was removed on postoperative day 7, and intensive swallowing rehabilitation was initiated. On postoperative day 10, the tracheostomy opening was sutured. The nasogastric feeding tube was removed on postoperative day 15 when sufficient autonomous oral feeding without aspiration was proved at a fibre-optic swallowing evaluation. The patient was discharged on postoperative day 20.

A definitive pathological evaluation confirmed a G2, p16-negative SCC of the left tongue base with extension over the midline, with perineural invasion, a residual cancer burden of 70%, extension to the left tonsil, and no lymph node metastases (0/27), thus defining ypT4a (DOI 11 mm) pN0. All resection margins were clear and over 5 mm.

**Figure 6:**
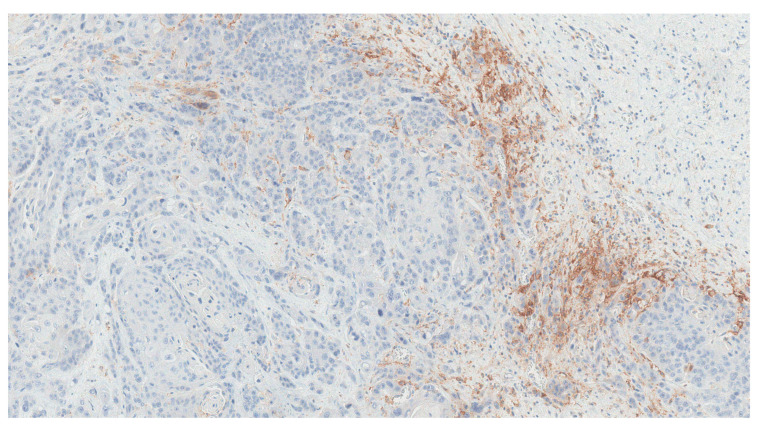
Programmed death-ligand 1 (PD-L1) expression in the surgical specimen, evaluated using the 22C3 PharmDx assay (Dako). The tumour was characterized by a low prevalence of immunoreactive cancer cells (<1%), and a moderate PD-L1 positive immune infiltrate, leading to a CPS equal to 10 (10×).

PD-L1 staining was assessed on the surgical specimen, revealing preserved PD-L1 expression in residual cancer cells (Figure 6).

Considering the pathological findings and final staging, the case was discussed again by the MDT, which resulted in a decision of maintenance therapy with pembrolizumab monotherapy.

**Figure 7:**
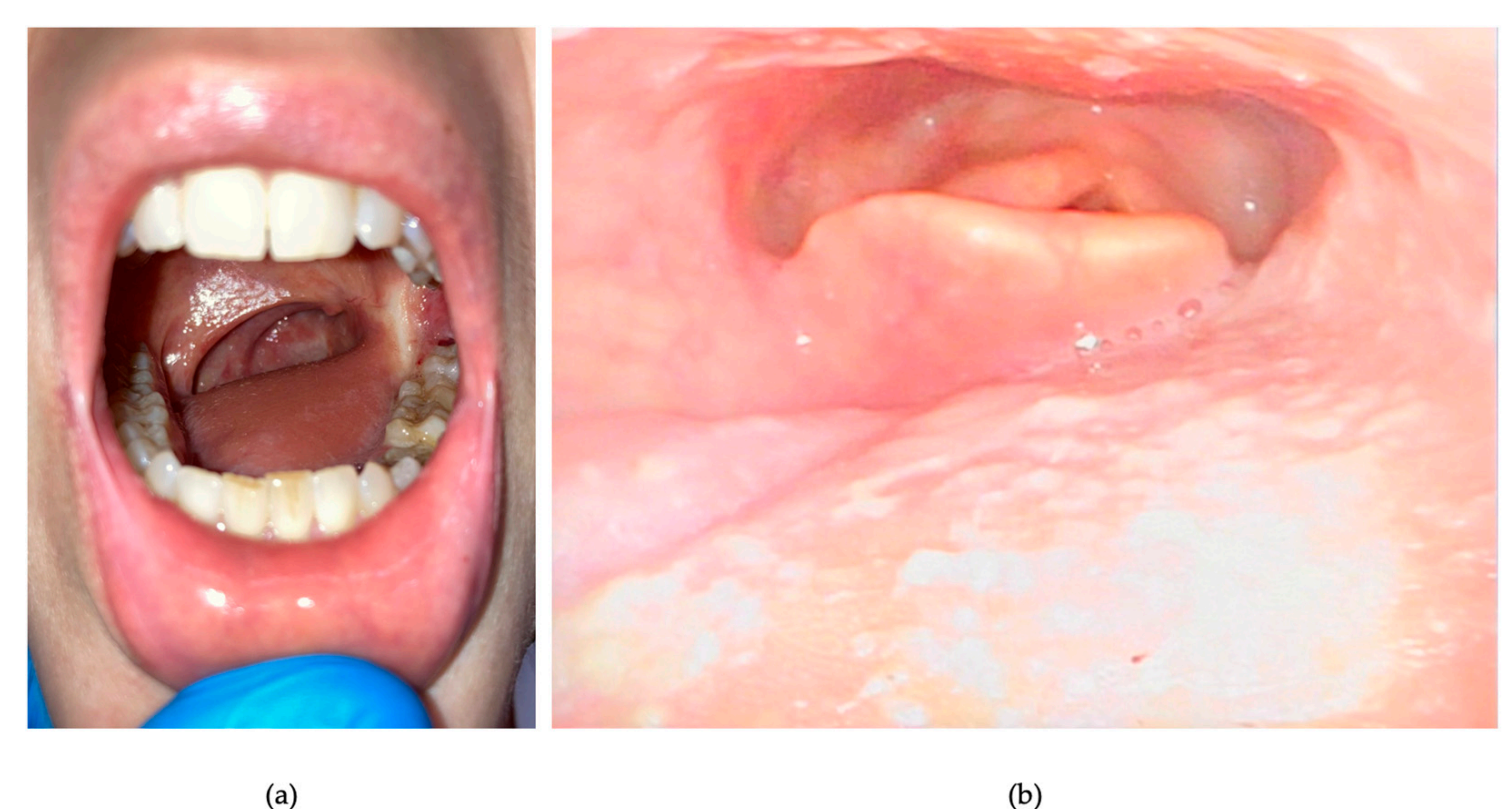
(**a**) Surgical outcome in oral cavity: the dorsal flap replaces the tongue and covers the left tonsillar fossa; (**b**) endoscopic view: excellent fitting of the neo-base of tongue; no significant pooling is observable.

Maintenance immunotherapy was initiated 40 days after surgery. Currently, the patient has completed three cycles of pembrolizumab (200 mg flat dose). The patient is in good general condition (Estern Cooperative Oncology Group Performance Status: 1). Exclusive oral feeding is adequate, with a stable body weight, and no clinical evidence of cancer relapse was found at the last clinical examination (103 days after surgery) (Figure 7).

## 3. Discussion

The reported case is an example of a second primary tumour (SPT) occurring in an irradiated field, with the only risk factor represented by previous RT in the head and neck district. Indeed, even though the overall risk of a new primary disease is enhanced in patients with a history of cancer, it is estimated that RT itself increases the risk from 1.2 to 3 times in the adult population [10,11]. Xiang et al., in a recent study involving more than 450,000 patients, found an incidence of 1.55 new malignancies per 100 patient-years [11]. In the head and neck district, incidence presents a wide range, from less than 0.3% to 15% [12,13], while the risk of RISCC after RT for NPC, as in the case presented herein, ranges from 0.82 to 5.6% [12,14,15]. Squamous cell carcinoma is the most frequently described histotype, followed by sarcomas (mainly osteosarcomas), even though some authors report an inverted ratio [16,17].

The most frequent subsite of occurrence of head and neck RISCC is the oral cavity, followed by the nasopharynx, larynx, and hypopharynx, while sarcomas are mostly located in the paranasal sinuses, skin, or temporal bone; in this case, the site of occurrence (base of tongue) is slightly unusual in the literature [17,18]. Ionising radiations present an intrinsic carcinogenic potential, but the mechanisms promoting the genesis of RISCC are still unclear. It has been supposed that an alteration in tissue oxygenation due radiation-induced hypoxia, hypovascularity, and hypocellularity may sustain the occurrence of a second malignancy [19]. Furthermore, it has been recently suggested that radiation-induced immune dysregulation may activate specific co-suppressor molecules called immune checkpoints (ICs); this could protect cancer cells against immune response [20]. In this light, immunotherapy could play a key role in the treatment of RISCCs, leading to clinical and pathological responses, as seen in the presented case.

**Figure 8:**
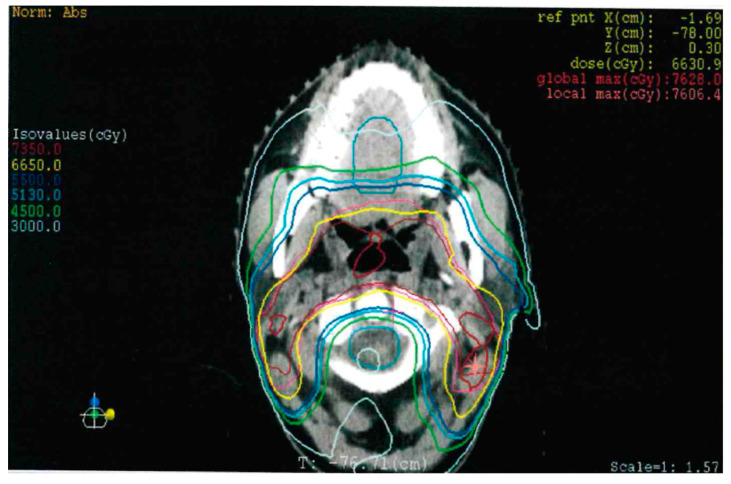
Axial image of the previous IMRT plan in correspondence of the actual disease, showing significative isodose curves.

Dose delivery, absorbed dose, and irradiation technique probably play a role in the genesis of the SPT and may contribute to its histology. Indeed, it is observed that the RISCCs usually occur in the lower dose areas of the irradiated field, while in the middle dose areas (above 48 Gy), sarcomas are more frequently represented [14,16,21,22]. In our specific case, however, the patient received an estimated dose of irradiation between 51.3 Gy and 66 Gy (Figure 8), suggesting the unpredictable nature of the SPT regardless of the dose delivered.

The different modalities of radiation, respectively, three-dimensional conformational radiation (3DCRT), intensity-modulated radiation therapy (IMRT), and proton beam radiation (PBRT), have been compared in the assessment of their potential toxic and carcinogenic roles; even though there are no harmonious results in the literature, it seems that no substantial differences are present between 3DCRT and IMRT, while a mild reduction in the risk of carcinogenesis for PBRT has been observed [11,23].

The latency period of RIM onset is unpredictable and widely ranges, from 3 years to more than 30 years after irradiation, with a median value of around 10 years [18,24]. Recently, Jang et al. retrospectively examined a cohort of RISCC patients previously treated for NPC, finding that those who received treatment before age 45 presented a median latency period significantly longer than the older arm (11 years vs. 8.5) [19]. In the present case, the SPT occurred after 13 years, in accordance with the median latency period reported in literature.

Diagnosis might be challenging since the awareness of the patient regarding early symptoms could be clouded by numb sensitivity due to the previous treatment; in addition, physical exams and radiological imaging could be hampered by anatomic post-actinic alterations and soft tissue fibrosis. Moreover, in our experience, we noticed that even tissue sampling for biopsies can be insidious since RISCCs often present as submucosal masses, spreading into stiff and chronically inflamed tissue with no clear surface alteration, forcing the practitioner to take an incisional biopsy in areas that are not always easy to access. In our patient, we needed to perform an inspection under general anaesthesia to ensure a diagnosis of persistence of disease after chemo-immunotherapy. Furthermore, the growth patterns of RISCCs tend to be less respectful of fascial planes and anatomical boundaries as a possible result of tissue fusion after RT [25].

Patients affected by RISCCs present worse survival rates than those affected by de novo SCC as the diagnosis is usually late and in an advanced stage, even though nodal involvement is less frequent than in de novo SCC; these patients are less suitable for surgery with radical intent, which represents the mainstay of treatment for RISCC, and they often cannot be candidate for full-dose adjuvant or salvage re-irradiation [24,26]. For RISCC, re-irradiation could lead to serious complications and severe late toxicity, such as osteoradionecrosis, fibrosis, severe dysphagia, and fatal bleeding, could reduce the survival advantages [27]. No specific studies on re-irradiation for RISCC can be found in the literature. Retrospective data on patients with RISCC of the tongue after RT for NPC show that the survival rate of patients not receiving surgery was lower than that of the surgically treated group [12]. Nonetheless, when surgery is performed with radical intention, there is no difference in survival rate between RISCC and de novo SCC [24].

Among the clinicopathological features of RISCC, a lower incidence of nodal involvement has been observed compared to de novo SCC: the reason may lie in the upheaval and ablation of lymphatic vessels as result of the previous radiation, preventing or reducing the lymphatic spreading of the SPT [28]. Similarly, in the reported experience we found no pathological lymph nodes, despite radiology suspecting bilateral nodal involvement. These findings may help in choosing whether to perform preventive neck dissection or reserve such treatment in cases of clinical, cytologically or histologically proven nodal metastasis; nevertheless, in our specific case, the administration of chemo-immunotherapy may have altered the final histologic findings; in fact, while pre- and post-treatment MRI showed unchanged bilateral suspect lymph nodes, pre-treatment PET/CT showed lymph node uptake in only one ipsilateral lymph node that lost uptake at post-treatment PET/CT. In this setting, a clinical study with nivolumab +/− tadalafil showed discordant pathologic treatment effects between the primary tumour and regional lymph node metastases [29]. Currently, available data (either prospective or retrospective) neither support nor limit the use of immune checkpoint inhibitors in RISCC patients. The patient under study was diagnosed with a locally advanced oropharyngeal SCC with clinical evidence of regional lymph node involvement. There is increasing evidence that pre-operative immunotherapy (i.e., the combination of anti-CTLA4 ipilimumab plus anti-PD1 nivolumab) induces major responses in primary tumours but not on regional nodes, as shown in the IMCISION clinical trial [30]. Therefore, we cannot exclude that the absence of lymph node involvement (pN0) observed after surgery may have been influenced by the synergy between chemotherapy and pembrolizumab. In this scenario, we still lack a standardised system to grade the immunotherapy response at a pathologic level. Nonetheless, pan-tumour scoring systems, which consider the residual tumour, necrosis, and regression bed, have been proposed in the literature [31].

There are no data available about the employment of chemo-immunotherapy for RT-induced HNSCC. Neoadjuvant systemic therapy has been widely examined in several trials for resectable HNSCC, mainly in the oral cavity, without evidence of an improvement in survival, while its role in ab initio unresectable tumours has never been confirmed [32,33,34,35]. Therefore, induction chemotherapy for locally advanced HNSCC should be considered standard of care only in the setting of organ preservation for laryngeal and hypopharyngeal SCCs.

Immunotherapy has been approved for unresectable recurrent and metastatic HNSCC. Recently, several trials examined the effects of neoadjuvant anti-PD-1 treatment in resectable cancers, with encouraging results [36,37,38,39]. In the presented case, the choice to administer a first-line systemic therapy had the purpose of obtaining a prompt clinical response, palliating cancer-related symptoms and assessing tumour responsiveness, thus giving the MDT the chance to predict the biological behaviour of the lesion while planning salvage surgery; this window of observation is typical of clinical trials assessing neoadjuvant treatment of HNSCC [39].

Due to the favourable response, the patient underwent surgery without suffering from limiting toxicities. Based on post-induction macroscopic extension, a subtotal glossectomy would have been feasible, but we decided to maintain the pre-induction intended resection margins, and definitive pathology revealed tumour extension over the midline, while this was no longer present at post-induction MRI and post-induction examination under narcosis.

During systemic treatment, the patient was clinically assessed weekly to rule out tumour progression. We wanted to avoid the risk of a critical progression towards the vallecula, making laryngeal preservation impossible. Since the patient was clinically and endoscopically responding, no additional early MRI was required.

Had the tumour completely responded to the systemic therapy (negative MRI, negative PET-CT, and negative biopsies), we would have faced a difficult scenario: deciding whether to proceed with surgery based on the pre-treatment tumour extension or not. Since complete pathological responses have been described following immunotherapy [29,39,40], and given that total glossectomy resulting in a complete pathological response would have been a dramatic occurrence, we probably would have continued with maintenance immunotherapy and reserved surgery in case of relapse.

## 4. Conclusions

RISCCs represent a rare and life-threatening long-term effect of RT. The wide range of the latency period and the unpredictability of the subsite make early diagnosis challenging. Surgery still represents the only effective therapy for these pathological entities, even though the previous treatment, the late diagnosis, and the high rate of postoperative complications can represent a real clinical challenge.

Further research is needed to better define the role of preoperative (chemo)immunotherapy and the potential clinical benefit of immune checkpoint inhibitors in RISCC patients.

## Data Availability

No new data were created or analysed in this study. Data sharing is not applicable to this article.
